# Extraction of Lycopene from Tomato Using Hydrophobic Natural Deep Eutectic Solvents Based on Terpenes and Fatty Acids

**DOI:** 10.3390/foods11172645

**Published:** 2022-08-31

**Authors:** Anastasia Kyriakoudi, Alexandros Tsiouras, Ioannis Mourtzinos

**Affiliations:** Laboratory of Food Chemistry and Biochemistry, Department of Food Science and Technology, School of Agriculture, Faculty of Agriculture, Forestry and Natural Environment, Aristotle University of Thessaloniki (AUTH), 54124 Thessaloniki, Greece

**Keywords:** hydrophobic natural deep eutectic solvents, lycopene, tomato, response surface methodology, Fourier transform-infrared spectroscopy

## Abstract

The present study proposes a green extraction approach for the recovery of lycopene from tomato fruits. Different hydrophobic natural deep eutectic solvents (HNADESs) based on terpenes (i.e., menthol and thymol) and fatty acids (i.e., decanoic acid and dodecanoic acid) were prepared at different molar ratios, characterised in terms of density, rheological properties, and Fourier transform-infrared (FT-IR) spectroscopy, and were examined for their effectiveness to extract lycopene from tomato. Response surface methodology (RSM) was employed to optimise the extraction parameters, namely duration (min) and solvent:solid ratio (*v*/*w*). Spectrophotometry and RP-HPLC-DAD were used in order to monitor the process efficiency. The combination of decanoic acid and dodecanoic acid was found to exhibit comparable extraction capacity to acetone. Taking into account that the HNADESs used in the present study are considered green, biodegradable and of low cost, the obtained carotenoid rich extracts are expected to be of use in industrial food applications.

## 1. Introduction

Lycopene (2,6,10,14,19,23,27,31-octamethyl-2,6,8,10,12,14,16,18,20,22,24,26,30-dotriacontatridecaene, C_40_H_56_), a bioactive lipid-soluble compound, belongs to the class of carotenoids which are one of the most well-known groups of natural colorants. Its de novo biosynthesis is observed in photosynthetic plants and some microorganisms whereas humans cannot synthesise it so they must include it in their diet [1]. The major edible source of lycopene is the fully ripe tomato fruits (ca. 90% of total carotenoids, followed by β-carotene and traces of lutein). Its levels in fresh tomatoes usually range between 1 to 10 mg lycopene/100 g fresh weight, depending on the variety, the stage of maturity as well as the cultivation practices [2]. Other dietary sources include watermelon, grapefruit, papaya, and guava [3].

Lycopene, either extracted from tomatoes or the fungus *Blakeslea trispora* or produced via chemical synthesis, has been authorised within the European Union as a food colouring agent (E160d) [4] in various food products [5]. Its industrial importance stems not only from its colouring attributes but also from its bioactive properties such as antioxidant, anticancer and immunomodulatory [1]. As the world is currently confronting the negative effects of the COVID-19 pandemic on global health, consumers are trying to protect themselves by adopting healthier nutritional habits in order to boost their immune system. This consumers’ need has led many food companies to commercialise innovative functional foods containing various bioactive compounds [6,7], such as lycopene. The commercial market value of lycopene was estimated to be $126 million in 2021 and is foreseen to reach $161 million by 2026 at an annual growth rate of 5.0% [8].

The constantly increasing lycopene’s demand for food, pharmaceutical and cosmeceutical applications, has led the scientific community to explore alternative approaches to extract it from natural sources. Recovery of lycopene can be affected by more than one factor, such as the means of extraction, the solvent, its location in the cells etc. Depending on the aim of the study, an array of techniques (e.g., ultrasound- or microwave-assisted extraction, supercritical fluid extraction etc.) and conventional organic solvents (e.g., hexane, acetone, ethanol, ethyl acetate etc.) as well as their mixtures, have been used so far for the extraction of lycopene [3,6]. Lately, the interest of the scientific community has focused on the use of alternative, green solvents such as the Deep Eutectic Solvents (DESs). The latter ones are defined as combinations of components that consist of a hydrogen bond donor (HBD) and a hydrogen bond acceptor (HBA) which form a network of hydrogen bonds resulting in a liquid with distinct characteristics, e.g., lower melting point, than that of the individual starting components [9]. DESs exhibit certain advantages such as biodegradability, biocompatibility, low cost, and easy preparation [10]. DESs that are synthesised by components of natural origin (e.g., plant metabolites) are called Natural Deep Eutectic Solvents (NADESs) [11], with the most common ones being composed of choline chloride, organic acids (e.g., acetic acid, malic acid, citric acid) and sugars (e.g., glucose, fructose, sucrose) [9]. Apart from hydrophilic NADESs, the use of hydrophobic natural deep eutectic solvents (HNADESs) has been recently emerged [10,12]. Different HNADESs consisting of quaternary ammonium salts or terpenes (e.g., menthol, thymol, geraniol, camphor, α-terpineol) with organic acids such as fatty acids [e.g., nonanoic acid, decanoic acid (capric acid), dodecanoic acid (lauric acid), oleic acid], have been reported in literature for the extraction of various hydrophobic bioactive molecules [13,14,15]. In this frame, HNADESs have been also used for the extraction of carotenoids from spirulina [16] and crude palm oil [17] as well as of individual carotenoids such as astaxanthin from the microalga *Haematococcus pluvialis* [18] and brown crab shell residues [19], lutein from the microalgae *Scenedesmus* sp. [20], β-carotene from pumpkin [21] and β-carotene and lycopene from fruit juices [22]. However, to the best of our knowledge information about the use of HDESs for the extraction of lycopene from tomato is limited and refers exclusively to tomato by-products [23,24,25].

In the present study, different HNADESs composed of terpenes, i.e., menthol and thymol, and/or fatty acids, i.e., capric acid and lauric acid (see Supplementary Materials, Figure S1), were prepared, physicochemically characterised and examined for their effectiveness to extract lycopene from tomato fruits for further potential applications based on its colouring as well as health-promoting properties. In this study, the whole tomato fruits were used as a natural source of lycopene, however, our proposed methodology could be also applied in tomato by-products (e.g., peels). The extraction conditions were further optimised by applying response surface methodology (RSM) [26]. UV-Vis spectrophotometry and RP-HPLC-DAD were employed to follow the effectiveness of the extraction process. Moreover, recovery of carotenoids from a HNADES extract based on their precipitation which is caused by the polarity switch of the eutectic mixture upon the addition of an anti-solvent, was also carried out, addressing the greatest challenge regarding the use of DESs, i.e., the recovery of the target compound(s) from the eutectic solvents [27,28].

## 2. Materials and Methods

### 2.1. Standards, Reagents and Solvents

Lycopene was purchased from TRC (Toronto Research Chemicals Inc., Toronto, ON, Canada) whereas β-carotene (99.7%) was from HPC Standards GmbH (Cunnersdorf, Germany). DL-Menthol (≥95%) and lauric acid (≥98%) were from Sigma-Aldrich (St. Louis, MO, USA). Capric acid (≥98%) was from TCI (Zwijndrecht, Belgium). Thymol (ca. 98%) was from Alfa Aesar (Kandel, Germany) and ammonia solution (25%, *w*/*v*) was from ChemLab (Zedelgem, Belgium). The solvents used were of the maximum required purity. Acetonitrile (>99.9%), acetone (>99.8%) and methanol (>99.8%) (Chem-Lab, Zedelgen, Belgium) used were HPLC grade. All other reagents used were of analytical grade.

### 2.2. Plant Material

Fresh tomato fruits from a commercial Greek variety were purchased from a local supermarket in Thessaloniki (Greece) (moisture content; 92%, *w*/*w*). To ensure a similar developmental stage, fruits (*n* = 10) of similar fresh weight (275 ± 22 g), size (8.2 ± 0.7 cm) and color parameters (L* = 41.32 ± 0.45, a* = 21.13 ± 0.94, b* = 22.67 ± 0.65), as determined using a Chromameter (Konica Minolta, CR-400 Series, Tokyo, Japan) calibrated with a white tile (L* = 96.9, a* = −0.04, b* = 1.84), were only selected. Before processing, tomatoes were washed with tap and ionised water and then dried with absorbent paper. After removal of the calyx and the seeds, they were homogenised in a Pulverisette 11 Knife Mill (Fritsch GmbH, Idar-Oberstein, Germany) for 20 sec at 6000 rpm and then freeze-dried using a HyperCOOL HC8080 freeze-dryer (Gyrozen Co., Ltd., Incheon, Korea) (−80 °C, 0.1 mbar) and stored at −20 °C until further analysis.

### 2.3. Preparation of HNADESs

HNADESs were prepared by mixing appropriate amounts of the terpenes DL-menthol or thymol as HBAs and certain fatty acids, namely capric acid and lauric acid, as HBDs, to obtain specific molar ratios as shown in Table 1. Moreover, mixtures of capric acid and lauric acid at different molar ratios were also examined. The mixtures were heated at 50 °C upon stirring (750 rpm) until transparent homogeneous liquids were obtained. In the case of thymol-based HNADESs, pale yellow transparent liquids were obtained. After cooling, the HNADESs were used either within the same day or stored at room temperature for further use.

### 2.4. Characterisation of HNADESs

The prepared HNADESs were physicochemically characterised in terms of density and rheological behaviour as well as by Fourier Transform-Infrared (FT-IR) Spectroscopy.

#### 2.4.1. Density

The density of the prepared HNADESs was measured using a Mettler Toledo Densito digital densitometer with a measuring range of 0.000–3.000 g/cm^3^ and accuracy of ±0.001 g/cm^3^. The instrument was calibrated using a standard solution of 0.9956 ± 0.0005 g/cm^3^ (30 °C).

#### 2.4.2. Rheological Behaviour

The rheological characteristics of the prepared HNADESs were evaluated using a Brookfield viscometer (DV-II+, Brookfield Engineering Labs, Inc., Middleboro, MA, USA). In all cases, viscosity measurements were carried out using spindle No 1 at a spindle speed of 5, 10, 20, 50 and 100 rpm. The viscosity values were fitted to the Power Law model as a function of shear rate as described by the equation 1 (Equation (1)) in order to calculate the flow behaviour (*n*) and flow consistency (b) indices.

µ_φ_ = (1/*n*)*^n^* · (4πN′)^*n*−1^ · b (1)

where µ_φ_; viscosity, *n*; flow behaviour index, b; flow consistency index, N′; rounds per second (rps).

#### 2.4.3. Fourier Transform-Infrared (FT-IR) Spectroscopy Analysis

The individual starting compounds as well as all the prepared HNADESs were analyzed using a FT-IR 6700 spectrometer (JASCO, Great Dunmow, UK). An aliquot of each sample was placed directly on an Attenuated Total Reflectance (ATR) sampling accessory MIRacle ™-Universal ATR (Pike Technologies, Madison, WI, USA) with a 3-Reflection Diamond/ZnSe Performance Crystal Plate. For solid starting materials (i.e., menthol, thymol, capric acid and lauric acid), constant pressure was applied by the pressure tool in order to achieve sufficient contact between the solid particles and the cell surface. For each sample, spectra were obtained in triplicate covering a range from 4000 to 400 cm^−1^ in the transmittance mode. For each spectrum, a total of 64 scans with 4 cm^−1^ resolution were acquired against a background obtained with a dry and clean cell. The original spectra were corrected for CO_2_, H_2_O and ATR effects, following this order, with the aid of Spectra Manager software (V.2.15.01, JASCO, Great Dunmow, UK). The corrected spectra were then subjected to smoothing and baseline correction using the same software.

### 2.5. Screening of HNADESs for the Extraction of Tomato Carotenoids

An aliquot (0.075 g) of the freeze-dried tomato sample was extracted with 3 mL of the prepared HNADESs (Table 1), with the aid of magnetic stirring (750 rpm) for 60 min with a sample:solvent ratio of 1:40 (*w*/*v*) at room temperature based on preliminary results (data not shown). All manipulations were carried out away from direct light to minimise photodecomposition of carotenoids throughout the analytical procedure. Successive extractions under the same experimental conditions using acetone as the extraction solvent was also carried out until the solid residue became colorless. An acetone extract was used as control in order to compare the extraction efficiency of the examined HNADESs with that of a conventional organic solvent. All extractions were performed in triplicate and the results were expressed as the mean value ± standard deviation.

### 2.6. Monitoring of Process Efficiency

The efficiency of extraction of tomato carotenoids and in particular of lycopene and β-carotene, was monitored by means of UV-Vis spectrophotometry and RP-HPLC-DAD analysis.

#### 2.6.1. UV-Vis Spectrophotometry

Absorption measurements at 473 nm were obtained in triplicate with a UV-1800 spectrophotometer (Shimadzu, Kyoto, Japan) equipped with glass cells (1 cm × 1 cm × 4 cm) for each extract after appropriate dilution (1:2, *v*/*v*) with methanol. Quantification of total carotenoids (mg/100 g fresh weight) was carried out with the aid of a proper calibration curve of lycopene (y = 0.2136x + 0.0046, R^2^ = 0.998, 0.1–10 ng/µL). Intra-day measurement repeatability was found to be satisfactory (CV% = 2.9 for mean mg total carotenoids/100 g fresh weight: 7.50, *n* = 7).

#### 2.6.2. RP-HPLC-DAD Analysis

Lycopene, β-carotene and total carotenoids were determined by RP-HPLC-DAD. The HPLC system consisted of a Marathon IV series HPLC pump (Rigas Laboratories, Thessaloniki, Greece), an injection valve with a 20 µL fixed loop (Rheodyne Cotaki, CA, USA) and a UV6000 LP diode array detector (DAD; Thermo Separation Products, San Jose, CA, USA). Separation was carried out isocratically on a Kromasil 100 C18 5 μm (250 × 4.6 mm i.d.) column (MZ Analysentechnik GmbH, Mainz, Germany) as described by Mantzouridou and Tsimidou [29]. Column temperature was set at 30 °C with the aid of a Timberline TL-50 controller and maintained as such in a TL-340 column heater. The elution system consisted of a mixture of acetone:acetonitrile (60:40, *v*/*v*). The flow rate was 1.2 mL/min. The injection volume was 20 µL. The analytical sample was prepared after proper dilution (1:2, *v*/*v*) with methanol and filtration through 0.45 μm PTFE filters (Frisenette, Knebel, Denmark). Chromatographic data were processed using the ChromQuest version 3.1.6 software (Thermo Electron Corporation, Beverly, CA, USA). Monitoring was in the range 380–600 nm. Identification of lycopene and β-carotene was achieved by comparing the retention times and spectral characteristics (absorption maxima) with those of available standards. Quantification of lycopene and β-carotene (mg/100 g fresh weight) was carried out by integration of the respective peaks at 473 and 453 nm with the aid of proper calibration curves (y = 41,067x − 57,750, R^2^ = 0.995, 20–495 ng/20 µL, *n* = 6 and y = 39,336x − 12,808, R^2^ = 0.999, 1–40 ng/20 µL, *n* = 5, respectively). Standard solutions of both lycopene and β-carotene were measured five times intra-day and analyzed on three consecutive days (inter-day) aiming to evaluate the repeatability of the method (% relative standard deviation, %RSD). The intra-day %RSD values were in the range 1.3–3.5 for lycopene and 1.5–3.9 for β-carotene. The inter-day values were in the range 1.5–4.6 and 1.9–4.9, respectively.

### 2.7. Experimental Design for the Selection of Extraction Parameters

The freeze-dried tomato sample was extracted with the aid of magnetic stirring for various time periods, at room temperature. Appropriate amount of freeze-dried sample (0.025–0.150 g) was added into a 10-mL amber glass vial, and then 3 mL of the most efficient HNADES as found during the screening process, were added. An unblocked full factorial central composite design (CCD) [26] was applied to examine two variables, i.e., duration of extraction (min) (X_1_) and solvent:solid ratio (*w*/*v*) (X_2_). Each one of the variables had five levels, namely: −a, −1 (low level), 0 (mid-level), +1 (high level), +a, with a being equal to 2^*n*/4^, where *n* = number of variables. For each one variable, the low and the high levels were chosen whereas the rest of the values derived from the equation given as a footnote in Table 2. For a total of thirteen experiments, that were set using the software Minitab 15.1.20.0 (Minitab, Inc., State College, PA, USA), there were five center points which were replicated in order to detect any deviation in linearity that may exist in the model (Table 3).

Polynomial response surfaces were fitted to the response, i.e., lycopene content (mg/100 g fresh weight) (CV% < 5, *n* = 5). Statistical analysis of the experimental data was carried out by RSM using the similar software. The second-order polynomial model was fitted to Equation (2):

Υ = β_0_ + β_1_X_1_ + β_2_X_2_ + β_11_X_1_^2^ + β_22_X_2_^2^ + β_12_X_1_X_2_ (2)

where Y is the predicted response, X_1_ and X_2_ are the levels of the coded variables, and β_0_, β_1_, …, β_12_ are the estimated coefficients, β_0_ being a scaling constant. The quality of the fit of the model was assessed by the coefficient of determination (R^2^) and the significance of each variable through F test and the lack of fit of the model. Coefficients with a *p* value < 0.05 were considered significant. Non statistically significant terms where omitted. The combinations of the optimal values of each variable that result in optimal responses were validated experimentally (*n* = 3) and compared to model prediction outcomes. The efficiency of extraction of lycopene from the tomato sample was examined by RP-HPLC-DAD analysis, as explained above.

### 2.8. Recovery of Carotenoids from a HΝADES Extract

A tomato extract rich in carotenoids was prepared using the mixture of Cap:Lau at a molar ratio of 1:2, with a solid:solvent ratio of 1:64 (*w*/*v*) with the aid of magnetic stirring for 1 h at room temperature. The extract was centrifuged (5000 rpm, 5 min) and the supernatant was collected. Deionised water (2 mL) was added to the supernatant resulting in the formation of two phases. Then, ammonia solution (25%, *w*/*v*) was added in excess at a ratio of 1:13 (*v*/*v*) and was thoroughly mixed for 2 min till obtaining a single hydrophilic phase due to in-situ solvent polarity switch [21,30]. The switched solution was stored in the dark for 72 h at room temperature. Samples were then subjected to centrifugation (7000 rpm, 15 min) and the precipitated carotenoids were collected and analyzed by RP-HPLC-DAD. The procedure was carried out in triplicate.

### 2.9. Statistical Analysis

Statistical comparisons of the mean values were performed by one-way ANOVA, followed by the multiple Duncan’s test (*p* < 0.05 confidence level) whereas comparisons between two mean values were performed by Independent Samples T-test using the IBM SPSS Statistics for Windows software, Version 27.0 (IBM Corp., Armonk, NY, USA).

## 3. Results and Discussion

### 3.1. Preparation and Characterisation of HNADESs

In the present study, fifteen different HNADESs composed of natural and edible components, i.e., terpenes (DL-menthol and thymol) and fatty acids (capric acid and lauric acid) were prepared at various molar ratios (Table 1). HNADESs composed exclusively of a combination of these fatty acids were also prepared since fatty acids can act simultaneously as HBAs and HBDs due to the presence of the OH group [10,28].

The prepared eutectic solvents were then physicochemically characterised considering that the composition of a DES has a great impact on its physicochemical properties that can affect its extraction efficiency. In particular, density is one of the most important physical properties of DESs since it can be used in thermodynamic models and process simulations that are needed for studying mass transfer, heat transfer etc. [31]. In the present study, the density of all of the prepared HNADESs was found to be lower than that of water and in particular in the range between 0.859 to 0.925 g/cm^3^ (Table 1), in contrast to the density of hydrophilic DESs which is usually higher than that of water (~1.15 g/cm^3^) [10] Although all the measured densities were similar, thymol-based eutectic mixtures presented higher densities than the respective menthol-based ones. The lower density (0.859 g/cm^3^) was observed for the HNADES consisting of a combination of capric acid and lauric acid at a molar ratio of 1:2. All the density values found in the present study are in line with literature reports for HNADESs composed of terpenes and/or fatty acids (e.g., [32,33]). The rheological properties of the prepared HNADESs were also examined in order to evaluate their efficiency as extraction solvents. The apparent viscosity of the all of the eutectic solvents prepared in the present study was found to be increasing along with shear rate (see Supplementary Materials, Figure S2), indicating a non-Newtonian, shear-thickening (i.e., pseudoplastic) behaviour. Only the mixture of Cap/Lau, 1:2, was found to be a Newtonian fluid as its apparent viscosity was found to be constant under different shear rates. The flow behaviour index (*n*) and the flow consistency index (b), calculated based on the Equation (1) (see Section 2.4.2), are presented in Table 1. The flow behaviour indices of all HNADESs were found to range between 1.203 to 2.251 except for that of Cap/Lau, 1:2, which was found to be 1.0. Moreover, HNADESs containing capric acid were found to present lower flow behaviour indices than the respective ones containing lauric acid. This seems to be linked to the alkyl-chain length, in line with literature reports according to which the viscosities of HDESs prepared with the same HBA tend to decrease along with a decrease of chain length of the HBD [10]. Moreover, a trend towards lower flow behaviour indices was observed for the thymol-based eutectic solvents compared to the respective menthol-based ones. The opposite trend is observed for their density values, as above mentioned. Similar observations have been also reported in literature for menthol- and thymol-based HDESs [33]. In general, hydrophobic deep eutectic solvents are reported to be less viscous compared to the hydrophilic ones, an attribute that enhances mass transfer and broadens their applications as extraction solvents [10].

**Figure 1:**
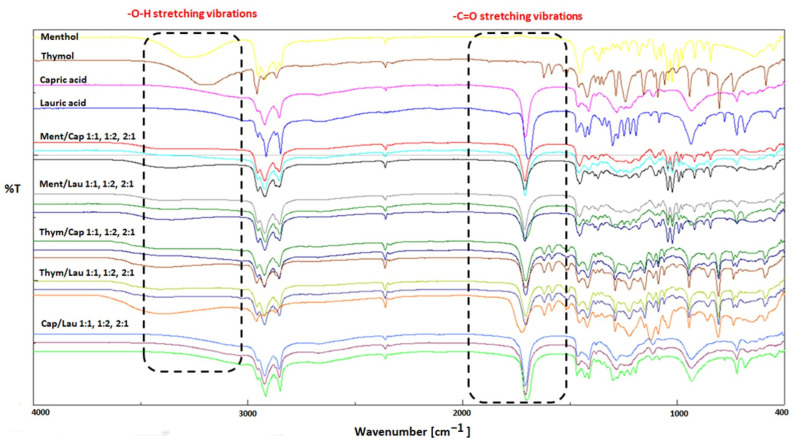
FT-IR spectra of pure menthol, thymol, capric acid and lauric acid as well as of the prepared HNADESs.

FT-IR spectroscopy was employed in order to examine the molecular interactions between the individual components of the prepared HNADESs. FT-IR is a useful technique to confirm the formation of hydrogen bonds between HBA and HBD. The FT-IR spectra of representative HNADESs as well as of their pure components (i.e., menthol, thymol, capric acid, lauric acid) are shown in Figure 1. In particular, the FT-IR spectrum of pure menthol showed a broad band at 3273 cm^−1^ assigned to the O-H bond, some distinctive absorptions at 2951 cm^−1^ and 2867 cm^−1^ attributed to the stretching vibrations of the bond C-H and an absorption at 1227 cm^−1^ attributed to C-O bond. Moreover, the absorptions at 1365 cm^−1^ and 1454 cm^−1^ are attributed to the bending of the C-H bonds of the CH_3_ (methyl) and CH_2_ (methylene) groups, respectively, that are present in menthol. On the other hand, the FT-IR spectrum of pure capric acid showed among others some distinctive absorptions at 2922 cm^−1^ attributed to C-H bonds, at 1702 cm^−1^ attributed to stretching vibrations of the C=O bond and at 1281 cm^−1^ attributed to C-O bond. Moreover, the bands at 1458 cm^−1^ and 1412 cm^−1^ correspond to the C-C bonds of the hydrocarbon chain. The examination of the FT-IR spectra of the prepared Ment/Cap HNADESs showed that the absorption of the O-H bond shifted to 3357 cm^−1^ whereas the C=O bond shifted to 1709 cm^−1^. Similar observations were also made for all the prepared HNADESs composed of combinations of menthol, thymol, capric acid and lauric acid as well as of the two fatty acids. Similar changes in these regions and especially shifts of the O-H and C=O bonds to higher wavelengths, have been reported in literature (e.g., [17,20]) and can be considered as an indication of the formation of a hydrogen-bond network between the individual components after the successful formation of the HNADESs.

### 3.2. Selection of the Most Efficient HNADES

The prepared HNADESs were examined for their efficiency to extract the major tomato carotenoids, i.e., lycopene and β-carotene. Extraction under the same experimental conditions using acetone as the extraction solvent was also carried out as reference. The extraction efficiency of the examined HNADESs is shown in Table 1. The most efficient HNADES for the extraction of tomato carotenoids was found to be Cap/Lau 1:2 followed by Thym/Lau 1:2 and Ment/Lau 1:1 whereas the least efficient ones were found to be the HNADESs consisting of menthol and capric acid. A typical RP-HPLC-DAD chromatogram of a tomato extract prepared using the Cap/Lau 1:2, eutectic mixture, is shown in Figure S3 of the Supplementary Materials. This eutectic mixture was found to have the lowest density and flow behaviour index among all the prepared HNADESs (Table 1). Even though menthol-based HNADESs have been shown to be efficient for the extraction of carotenoids from various sources e.g., crude palm oil (i.e., DL-menthol/lauric acid, 2:1) [17], tomato processing by-products (i.e., DL-menthol/lactic acid, 8:1) [25] etc., in the present study a fatty acid-based HNADES was found to be the most efficient one. A HNADES consisting of two fatty acids, i.e., caprylic acid and capric acid at a molar ratio of 3:1 has been also reported to be the most efficient for the recovery of β-carotene from pumpkin [21]. Similar observations have been also made for the extraction of carotenoids from spirulina [16] where the most efficient eutectic solvents were found to be those consisting of mixtures of fatty acids, namely, octanoic acid, nonanoic acid, decanoic acid or dodecanoic acid. Based on all the above, the Cap/Lau 1:2 eutectic mixture was selected for further optimisation of the extraction process.

### 3.3. Optimisation of Extraction Conditions

#### 3.3.1. Selection of Extraction Conditions Using the RSM

A CCD was applied to select the experimental conditions for the examination of the effect of duration of extraction (X_1_) and solvent:solid ratio (X_2_) on total tomato carotenoids content, with lycopene accounting for more than 90% of total carotenoids in tomato. The statistical model fitted to the data for the responses (Table 3) allowed assessment of factor interactions and enabled optimisation of the extraction conditions. Verification of the model was then tested experimentally.

#### 3.3.2. Model Fitting for Lycopene Content (mg/100 g fresh weight)

Experimental results for lycopene content (mg/100 g fresh weight) were analyzed by ANOVA (see Supplementary Materials, Table S1) to test the validity of the model on the basis of *F*-test values (see Section 2.7). The model, in terms of actual factor values (Table 2), fitted for the response is shown by Equation (3). The experimental values (mg lycopene/100 g fresh weight) were fitted to a full second-order polynomial model, which was reduced by omitting the insignificant terms (*p* > 0.05) (see Supplementary Materials, Table S2).

Lycopene content (mg/100 g fresh weight) = −4.9308 + 0.1897X_2_ − 0.0019X_1_^2^ − 0.0015X_2_^2^ (3)

The ANOVA revealed that there is no significant lack of fit (*p* = 0.366) of the model. Additionally, the coefficient of determination (R^2^) was 0.855 which means that more than 85% of the variability in the response could be explained by the model. Consequently, Equation (3) give an adequately describe the experimental data and is appropriate for use in the optimisation of lycopene content (mg/100 g fresh weight) response.

#### 3.3.3. Main Effects of Factors and Interactions to the Experimental Response for Lycopene Content (mg/100 g Fresh Weight)

Results of the statistical analysis (see Supplementary Materials, Table S2) demonstrated that the variable solvent:solid ratio (X_2_) had a positive and significant linear effect (at 95% confidence level). Its quadratic effect (X_2_X_2_) was also significant but negative. The linear term of the duration of extraction (X_1_) showed non-significant effects to the lycopene content (mg/100 g fresh weight) values whereas its quadratic effect (X_1_X_1_) was found to be significant.

**Figure 2:**
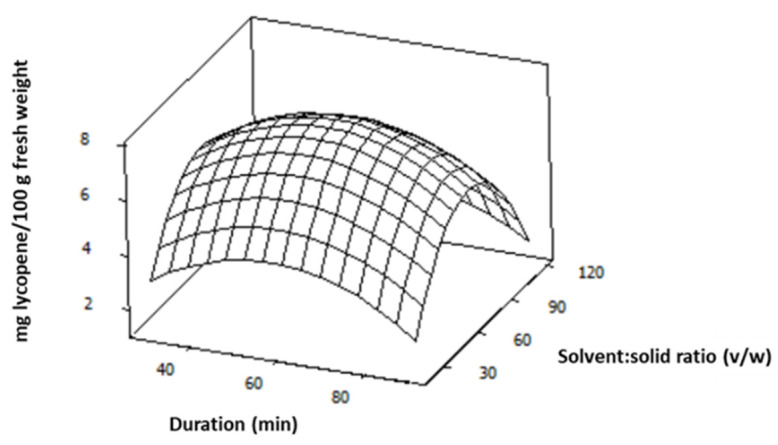
Surface plot for lycopene content (mg/100 g fresh weight) affected by duration of extraction (min) and solvent:solid ratio (*v*/*w*).

The fitted polynomial equation (Equation (3)) was expressed as a response surface plot (Figure 2) to visualise the correlation of lycopene content response and the experimental levels of each variable (X_1_, X_2_) as well as to determine the optimum conditions of the extraction. The plot shows that higher lycopene content values were observed by increasing solvent:solid ratio (*v*/*w*) up to the middle level. The characteristic curvature of the response surface exemplifies the negative quadratic effect of this factor to lycopene content values, indicating that this response is not favored by high solvent:solid ratios. Moreover, based on this plot, keeping solvent:solid ratio constant at its middle level, a maximum point can be observed at the middle level of duration of extraction, indicating that the response is not favored by prolonged extraction duration probably due to carotenoids degradation.

#### 3.3.4. Predicted and Verified Optimum Conditions for Lycopene Content (mg/100 g Fresh Weight)

The fitted model for Equation (3) was employed to predict optimum values of the variables. The optimum combination of the duration of extraction and the solvent:solid ratio for the recovery of lycopene was 62 min and 64:1 (*v*/*w*), respectively. The predicted content of lycopene (7.99 mg/100 g fresh weight) fits well with the average experimental value (7.90 ± 0.15 mg/100 g fresh weight, *n* = 3).

### 3.4. Application of Selected Extraction Conditions to Tomato Fruit Samples

The optimum extraction conditions were applied to five ripe tomato samples of different Greek varieties. The data are shown in Table 4 and confirm all of the above findings. The recovery of lycopene under the optimum extraction conditions using the mixture of Cap/Lau 1:2 was found to be comparable to that using acetone as the extraction solvent.

### 3.5. Recovery of Carotenoids from a HΝADES Extract

Based on their natural origin, NADES extracts do not require the removal of the solvent as well as further purification steps and therefore they could be used directly for food, pharmaceutical or cosmeceutical applications [16,34]. However, there is a growing interest in the scientific community towards methods for the recovery of the compound(s) of interest from eutectic mixtures as well as solvent recycling [27]. Such methods include liquid-liquid extraction, solid-phase extraction using resins or molecular sieves, ultrafiltration, and precipitation by addition of anti-solvents [35]. In the present study, the potential of fatty acids to act as switchable solvents, i.e., solvents that are able to alter their nature from hydrophobic to hydrophilic and vice versa, was exploited in order to recover tomato carotenoids from a HNADES extract, as it was previously reported in the case of carotenoids from pumpkin [21]. Fatty acids have been reported to become water soluble when they react with an amine through forming fatty acid-amine ion pair complexes via their carbonyl groups. Such solvents with switchable polarity offer advantages over traditional ones such as the possibility of solvent recycling that reduces the cost of the process [36].

**Figure 3:**
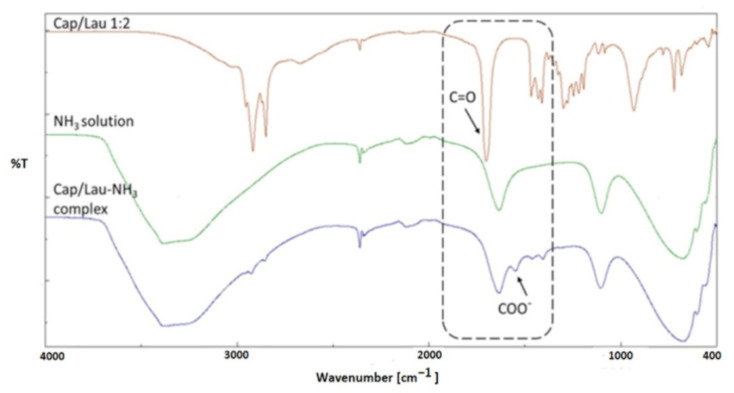
FT-IR spectra of the pure Cap/Lau 1:2 eutectic mixture, ammonia solution as well as Cap/Lau-NH_3_ complex.

In the present study, water addition to a tomato extract prepared with the most efficient fatty acid-based eutectic mixture, i.e., Cap/Lau 1:2, caused the formation of two phases due to different polarities of the mixed media. The subsequent addition of ammonia solution caused an increase of the pH resulting in the switch of the polarity of the HNADES extract causing the two phases to start mixing until only one phase was observed. This could be considered as an indication of the in-situ switch of the fatty acid-based HNADES. The HNADES-amine complex was studied using FT-IR. Figure 3 shows the FT-IR spectra of the pure Cap/Lau 1:2 eutectic mixture and ammonia solution as well as of the formed Cap/Lau-NH_3_ complex. It can be observed that after complexing with the amine solution, the peak at 1702 cm^−1^, which corresponds to the carbonyl bond of the fatty acids of the eutectic mixture, was shifted to 1543 cm^−1^ indicating the formation of carboxylate. Similar observations have also been made in the case of octanoic acid complexing with poly(oxypropylene) diamine [36].

After ca. 72 h, precipitation of carotenoids was observed due to their insolubility in the switched polarity media. RP-HPLC-DAD analysis of the precipitated carotenoids revealed that 36.9 ± 1.3% of the initial total carotenoids content were recovered, with the predominant carotenoid being lycopene. A recovery of 41.97% has been reported for curcuminoids from a choline chloride-based DES turmeric extract employing an anti-solvent precipitation technique [37] whereas 90% of carotenoids have been recovered from a pumpkin extract using a switchable-hydrophilicity eutectic solvent system [21]. It is worth mentioning that the switched extract can be either switched back with various approaches including bubbling with CO_2_, that causes the dissociation of the formed ion pair complex [36], or temperature adjustment (thermo-switchable DESs) [38] or it could be used as such for the extraction of hydrophilic compounds from tomato as well.

## 4. Conclusions

In conclusion, a fatty acid based HNADES was found to be the most efficient in extracting lycopene from tomato as a green alternative to conventional organic solvents. Taking into account that the HNADESs used in the present study are of natural origin, the obtained extracts could be directly incorporated in food, pharmaceutical or cosmeceutical preparations. However, toxicity studies of HNADEs extracts are of outmost importance prior to industrial applications. If isolated fractions are required, the target compounds can be recovered through polarity switch of the eutectic mixture. Further research is required in order to optimise the recovery process parameters, e.g., temperature, HNADES extract:ammonia solution ratio, duration etc. towards increasing the recovery yield. Our proposed methodology could be also applied in tomato by-products (e.g., peels).

## Figures and Tables

**Table 1:** Abbreviations, molar ratios and physicochemical characterisation of the prepared HNADESs, and tomato carotenoids content (mg/100 g fresh weight), as determined by RP-HPLC-DAD and UV-Vis spectrophotometry after extraction with different HNADESs during the screening process.

HBA	HBD	Abbreviation	Molar Ratio	Density *	Flow Behaviour Index (*n*) *	Flow Consistency Index (b) *	RP-HPLC-DAD			UV-Vis
							Lycopene Content (473 nm) **^,^***	β-Carotene Content (453 nm) **^,^***	Total Carotenoids Content (473 nm) **^,^***^,#^	Total Carotenoids Content (473 nm) **^,^***^,#^
							mg/100 g Fresh Weight			
Menthol	Capric Acid	Ment/Cap	1:1	0.877	1.259	0.016	2.73 ± 0.07 ^c^	0.17 ± 0.01 ^e^	2.97 ± 0.06 ^c^	2.55 ± 0.03 ^c^
		Ment/Cap	1:2	0.879	1.593	0.009	1.34 ± 0.04 ^b^	0.10 ± 0.01 ^c^	1.46 ± 0.05 ^a^	1.32 ± 0.02 ^a^
		Ment/Cap	2:1	0.876	1.203	0.020	1.06 ± 0.08 ^a^	0.08 ± 0.01 ^b^	1.19 ± 0.08 ^a^	1.07 ± 0.01 ^a^
Menthol	Lauric Acid	Ment/Lau	1:1	0.881	2.051	0.007	4.93 ± 0.56 ^g^	0.13 ± 0.03 ^d^	5.39 ± 0.64 ^g^	4.97 ± 0.01 ^e^
		Ment/Lau	1:2	0.876	2.201	0.006	2.89 ± 0.28 ^c^	0.07 ± 0.02 ^d^	3.18 ± 0.33 ^c, d^	2.89 ± 0.04 ^c^
		Ment/Lau	2:1	0.887	2.251	0.005	1.44 ± 0.02 ^b^	0.10 ± 0.02 ^c^	2.47 ± 0.77 ^b^	1.99 ± 0.02 ^b^
Thymol	Capric Acid	Thym/Cap	1:1	0.909	1.531	0.008	3.40 ± 0.18 ^e^	0.19 ± 0.01 ^g^	3.64 ± 0.24 ^d^	3.54 ± 0.06 ^d^
		Thym/Cap	1:2	0.918	1.549	0.008	4.58 ± 0.06 ^f^	0.19 ± 0.03 ^g^	5.28 ± 0.66 ^f, g^	4.97 ± 0.05 ^e^
		Thym/Cap	2:1	0.925	1.540	0.008	4.42 ± 0.17 ^f^	0.24 ± 0.01 ^h^	4.81 ± 0.23 ^e, f^	4.76 ± 0.05 ^e^
Thymol	Lauric Acid	Thym/Lau	1:1	0.912	2.055	0.005	4.53 ± 0.37 ^f^	0.25 ± 0.03 ^i^	4.88 ± 0.44 ^e, f^	4.69 ± 0.02 ^e^
		Thym/Lau	1:2	0.893	1.384	0.020	5.41 ± 0.69 ^i^	0.19 ± 0.01 ^g^	5.87 ± 0.75 ^i^	5.58 ± 0.04 ^f^
		Thym/Lau	2:1	0.911	2.094	0.005	5.15 ± 0.12 ^h, i^	0.28 ± 0.05 ^j^	5.66 ± 0.17 ^g, h^	5.49 ± 0.03 ^f^
Capric acid	Lauric Acid	Cap/Lau	1:1	0.881	1.355	0.012	2.98 ± 0.06 ^c, d^	0.18 ± 0.03 ^f^	3.32 ± 0.07 ^d^	3.01 ± 0.13 ^d^
		Cap/Lau	1:2	0.859	1.000	0.008	7.51 ± 0.10 ^j^	0.32 ± 0.05 ^k^	8.04 ± 0.10 ^j^	7.74 ± 0.07 ^g^
		Cap/Lau	2:1	0.865	1.547	0.024	3.19 ± 0.04 ^d^	0.17 ± 0.01 ^e^	3.52 ± 0.04 ^d^	3.45 ± 0.07 ^d^
Acetone							8.15 ± 0.14 ^k^	0.37 ± 0.01 ^l^	8.86 ± 0.17 ^k^	8.75 ± 0.03 ^h^

* Results are expressed as the mean value of two independent measurements. ** Different lowercase letters as superscripts within the same column for each HNADES differ significantly according to Duncan’s test at *p* < 0.05. *** Results are expressed as the mean value of three independent experiments ± standard deviation. ^#^ Expressed as lycopene.

**Table 2:** Levels of independent variables in actual and coded values.

Symbols	Factors			Level		
				Coded value *		
		−a **	−1	0	+1	+a **
				Actual value		
Χ_1_	Duration (min)	30	41	60	81	90
Χ_2_	Solvent:solid ratio (*v*/*w*)	20	35	70	105	120

* $\mathrm{CodedValue}=\frac{\mathrm{ActualValue}\;-\frac{\left(\mathrm{HighLevel}+\mathrm{LowLevel}\right)}{2}}{\frac{\mathrm{HighLevel}\;-\;\mathrm{LowLevel}}{2}}$. ** Level a represents the distance of a star point to the center point in a CCD and is equal to 2^*n*/4^, where *n* = number of variables.

**Table 3:** Experimental design and responses of the dependent variable expressed as mg lycopene/100 g fresh weight.

Run.	Duration (min)	Solvent:Solid Ratio (*v*/*w*)	Lycopene Content (mg/100 g Fresh Weight) (*n* = 3)
	X_1_	X_2_	
1	60	20	4.09 ± 0.32
2	60	70	7.87
3	60	70	8.14
4	30	70	5.12 ± 0.51 *
5	90	70	6.45 ± 0.61 *
6	60	70	8.09
7	41	35	5.87 ± 0.15
8	41	105	2.38 ± 0.45
9	81	105	3.69 ± 0.16
10	81	35	4.99 ± 0.12
11	60	120	3.04 ± 0.34
12	60	70	8.02
13	60	70	7.41

* *n* = 5.

**Table 4:** Application of the optimised extraction conditions (i.e., duration; 62 min, solvent:solid ratio; 64:1, *v*/*w*) to five randomly selected ripe tomato samples (*n* = 3).

Sample	Extraction Conditions *	Lycopene Content **	β-Carotene Content **	Total Carotenoids Content **
1	(a)	10.88 ± 0.57 ^a^	0.33 ± 0.01 ^a^	11.91 ± 0.58 ^a^
	(b)	11.01 ± 0.69 ^a^	0.35 ± 0.02 ^a^	12.44 ± 0.73 ^b^
2	(a)	14.92 ± 0.33 ^a^	0.91 ± 0.11 ^a^	15.98 ± 0.17 ^a^
	(b)	15.04 ± 0.34 ^a^	0.92 ± 0.10 ^a^	16.53 ± 0.42 ^b^
3	(a)	11.68 ± 0.47 ^a^	0.69 ± 0.04 ^a^	12.48 ±0.55 ^a^
	(b)	11.27 ± 0.76 ^a^	0.65 ± 0.06 ^a^	12.09 ± 0.82 ^a^
4	(a)	11.13 ± 0.50 ^a^	0.59 ± 0.01 ^a^	11.82 ± 0.49 ^a^
	(b)	10.24 ± 0.56 ^b^	0.53 ± 0.03 ^a^	10.88 ± 0.59 ^a^
5	(a)	11.82 ± 0.35 ^a^	0.65 ± 0.06 ^a^	12.59 ± 0.35 ^a^
	(b)	11.01 ± 0.74 ^a^	0.61 ± 0.03 ^a^	11.73 ± 0.76 ^a^

* Extraction conditions: (a) Cap/Lau, 1:2; and (b) acetone; ** Different lowercase letters as superscripts within the same column for each sample differ significantly according to Independent Samples *t*-test at *p* < 0.05.

## Data Availability

Data is contained within the article or Supplementary Material.

## Supplementary Materials

The following supporting information can be downloaded at: https://www.mdpi.com/article/10.3390/foods11172645/s1, Figure S1: Chemical structures of menthol, thymol, capric acid and lauric acid; Figure S2: Effect of shear rate on apparent viscosity of the prepared HNADESs; Figure S3: RP-HPLC-DAD chromatogram at 473 nm of a tomato extract prepared with Cap/Lau 1:2 eutectic mixture (the UV spectra corresponding to the peaks attributed to lycopene and β-carotene, respectively, are shown as inserts); Table S1: Analysis of variance of lycopene content (mg/100 g fresh weight) values; Table S2: Estimated regression coefficients and significance (*p*) values for lycopene content (mg/100 g fresh weight) after using coded values of factors.
