# Supplementary material for: Extraction of Lycopene from Tomato Using Hydrophobic Natural Deep Eutectic Solvents Based on Terpenes and Fatty Acids
Source: Foods. 2022 Aug 31;11(17):2645. doi: 10.3390/foods11172645 (PMC9455282; doi:10.3390/foods11172645)
Supplement: Supplementary file 1 [file foods-11-02645-s001.zip › foods-1834301-supplementary.pdf]

Supplementary Material

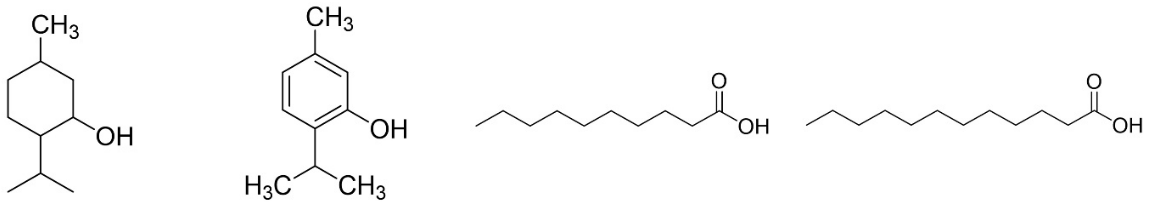

Menthol Thymol Capric acid Lauric acid

Figure S1. Chemical structures of menthol, thymol, capric acid and lauric acid.

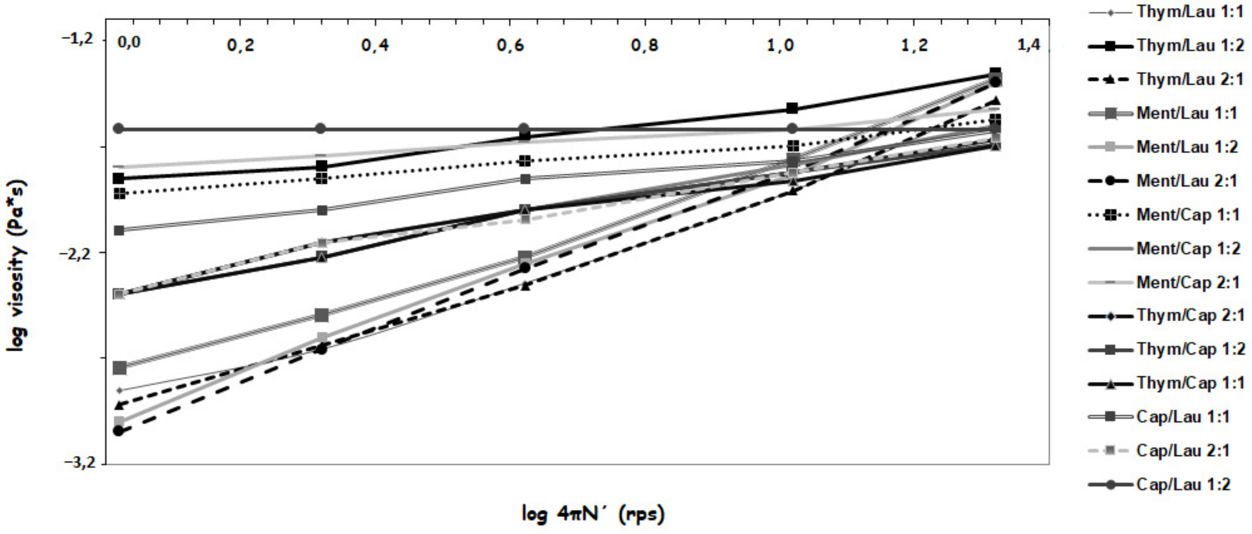

Figure S2. Effect of shear rate on apparent viscosity of the prepared HNADESs.

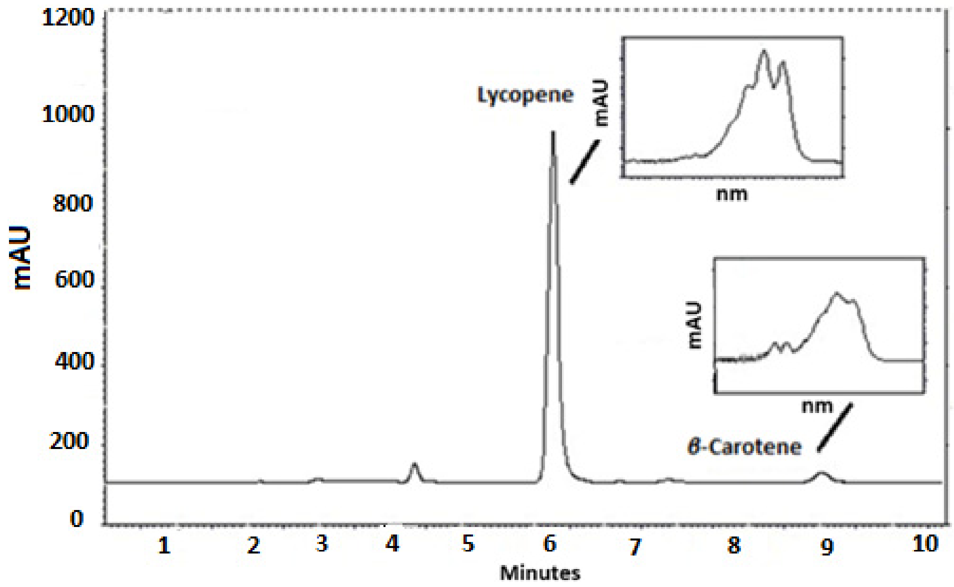

Figure S3. RP-HPLC-DAD chromatogram at 473 nm of a tomato extract prepared with Cap/Lau 1:2 eutectic mixture (the UV spectra corresponding to the peaks attributed to lycopene and β-carotene, respectively, are shown as inserts).

Table S1. Analysis of variance of lycopene content (mg/100 g fresh weight) values.

	Source	F-value [a]	P-value
	mg lycopene/100 g fresh weight (R^2 = 0.855)		
Regression		8.24	0.008
	Linear	7.42	0.019
	Square	19.73	0.001
	Interaction	0.10	0.760
Lack of fit		1.39	0.366

[a] F-test values are significant at the 5% level.

Table S2. Estimated regression coefficients and significance (*P*) values for lycopene content (mg/100 g fresh weight) after using coded values of factors.

Term	Coefficient	*P*-value
mg lycopene/100 g fresh weight		
Constant	-4.9308	0.240
Duration of extraction	0.2081	0.066
Solvent:solid ratio	0.1897	0.007
Duration of extraction x Duration of extraction	-0.0019	0.033
Solvent:solid ratio x Solvent:solid ratio	-0.0015	0.001
Duration of extraction x Solvent:solid ratio	0.0002	0.760
